# Supplementary material for: Genotoxic stress and viral infection induce transient expression of APOBEC3A and pro-inflammatory genes through two distinct pathways
Source: Nat Commun. 2021 Aug 13;12:4917. doi: 10.1038/s41467-021-25203-4 (PMC8363607; doi:10.1038/s41467-021-25203-4)
Supplement: Supplementary file 1 — Supplementary Information [file 41467_2021_25203_MOESM1_ESM.pdf]

Supplementary information for

Genotoxic Stress and Viral Infection Induce Transient Expression of APOBEC3A and Pro-Inflammatory Genes Through Two Distinct Pathways

Sunwoo Oh, Elodie Bournique, Danae Bowen, Pégah Jalili,

Ambrocio Sanchez, Ian Ward, Alexandra Dananberg, Lavanya Manjunath,

Genevieve P. Tran, Bert L. Semler, John Maciejowski, Marcus Seldin & Rémi Buisson

Supplementary Figure 1

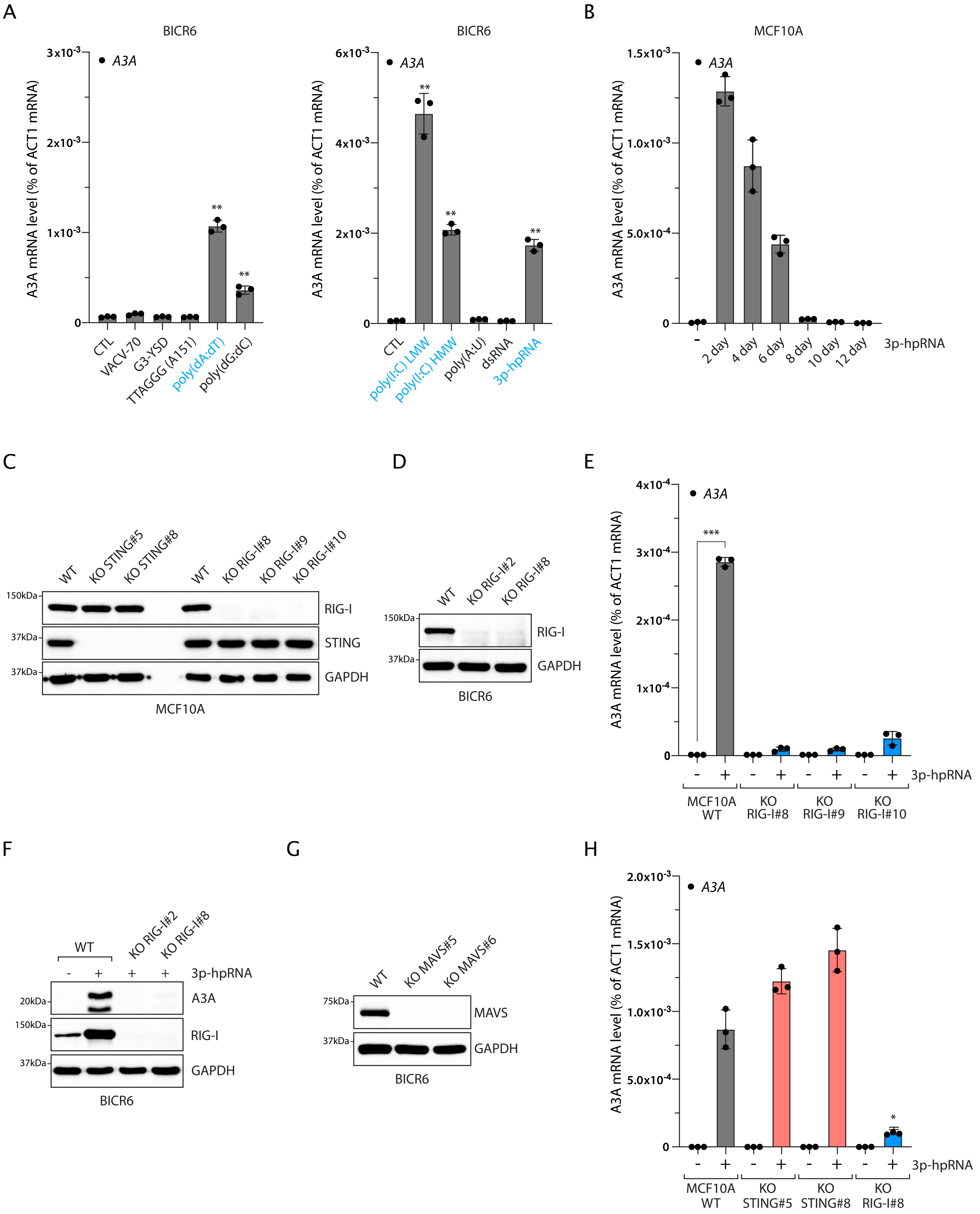

Supplementary Figure 1: A. Indicated DNA (200 ng/mL) or RNA (100 ng/mL) oligonucleotides were transfected in BICR6 cells. A3A mRNA level was monitored by RT-qPCR 16h after transfection. Mean values +/- S.D. (n = 3). ** *P* < 0.01 (two-tailed Welch t test). Known RIG-I agonist oligonucleotides are labeled in blue. **B.** MCF10A cells were transfected with 3p-hpRNA (40 ng/mL) and A3A mRNA level was monitored at the indicated time Mean values +/- S.D. (n = 3) **C.** STING and RIG-I protein levels in MCF10 WT, STING KO or RIG-I KO cells were analyses by western blot using STING and RIG-I antibodies. **D.** BICR6 WT and RIG-I KO cell lysates were analyzed by Western Blot using the indicated antibodies. **E.** A3A expression following 3p-hpRNA transfection in MCF10A WT or RIG KO cells. Mean values +/- S.D. (n = 3). *** *P* < 0.001 (two-tailed Welch t test). **F.** The A3A protein levels was monitored in BICR6 cells or indicated knockout cell lines 24h after 3p-hpRNA transfection. Mean values +/- S.D. (n = 3) **G.** BICR6 WT and MAVS KO cell lysates were analyzed by Western Blot using the indicated antibodies. **H.** A3A expression following 3p-hpRNA transfection in MCF10A WT, STING KO, and RIG-I KO cells. Mean values +/- S.D. (n = 3). Source data are provided as a Source Data file.

Supplementary Figure 2

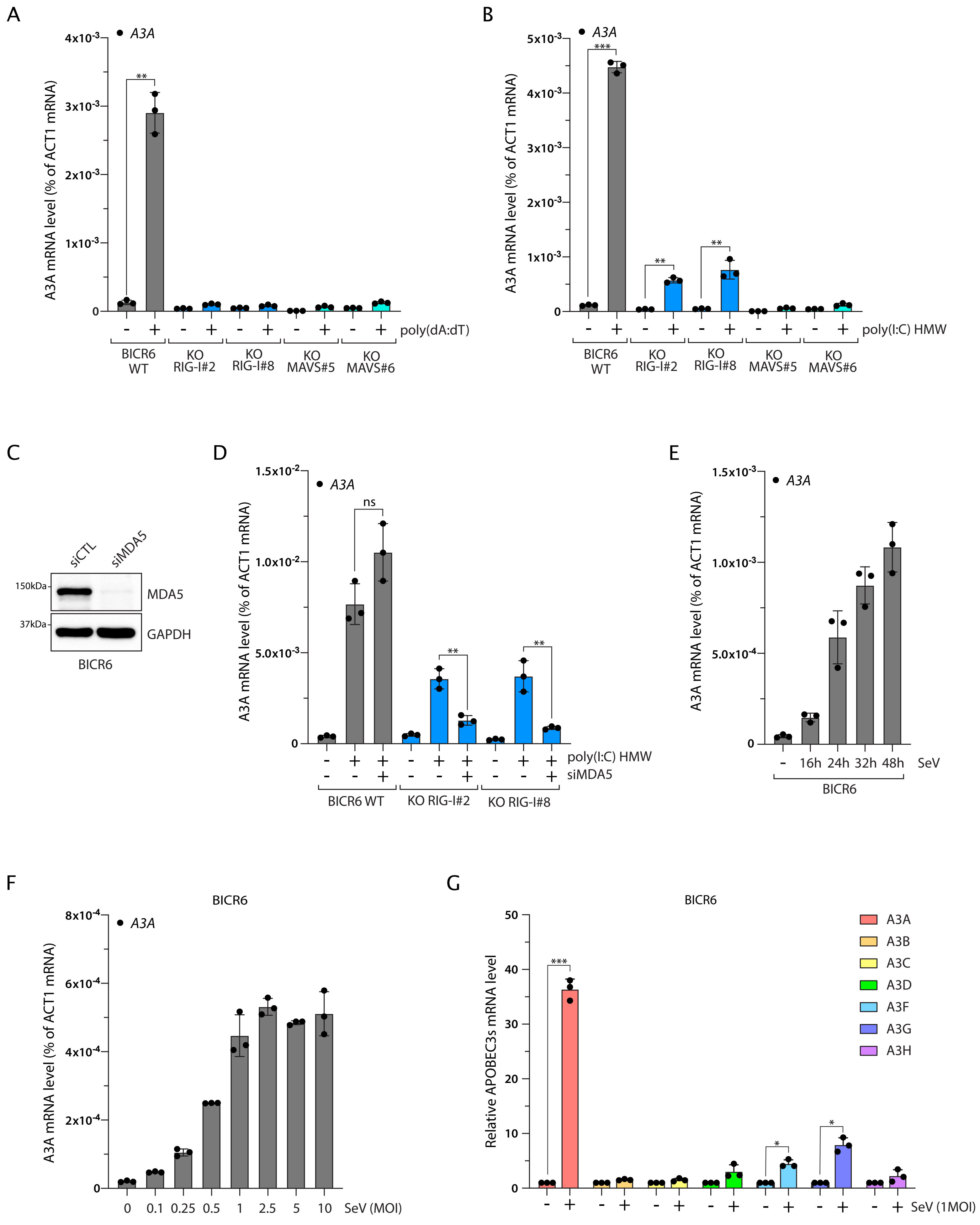

Supplementary Figure 2: A-B. Quantification of the A3A mRNA level 16h after poly(dA:dT) transfection (200 ng/mL) or poly(I:C) HMW (50 ng/mL) in BICR6 WT, RIG-I KO, or MAVs KO cells. Mean values +/- S.D. (n = 3). ** $P < 0.01$, *** $P < 0.001$ (two-tailed Welch t test). **C.** BICR6 cells were transfected with control or MDA5 siRNA. Cell lysates were analyzed by Western blot using the indicated antibodies. **D.** **I**ndicated BICR6 cell lines were transfected with siCTL or siMDA5 36h before transfection with poly(I:C) HMW. 16h after poly(I:C) HMW transfection, cells were collected and the A3A mRNA level was quantified by RT-qPCR. Mean values +/- S.D. (n = 3). ns: not significant ($P > 0.05$), ** $P < 0.01$ (two-tailed Welch t test). **E.** BICR6 cells were infected with SeV (1 MOI) for the indicated time and A3A expression level was determined by RT-qPCR. Mean values +/- S.D. (n = 3). **F.** BICR6 cells were infected with SeV for 24h at the indicated MOI and A3A expression level was determined by RT-qPCR. Mean values +/- S.D. (n = 3). **G.** BICR6 cells were infected with SeV for 24h and A3A, A3B, A3C, A3D, A3F, A3G, and A3H expression level was determined by RT-qPCR. Mean values +/- S.D. (n = 3). * $P <0.05$, *** $P < 0.001$ (two-tailed Welch t test). Source data are provided as a Source Data file.

Supplementary Figure 3

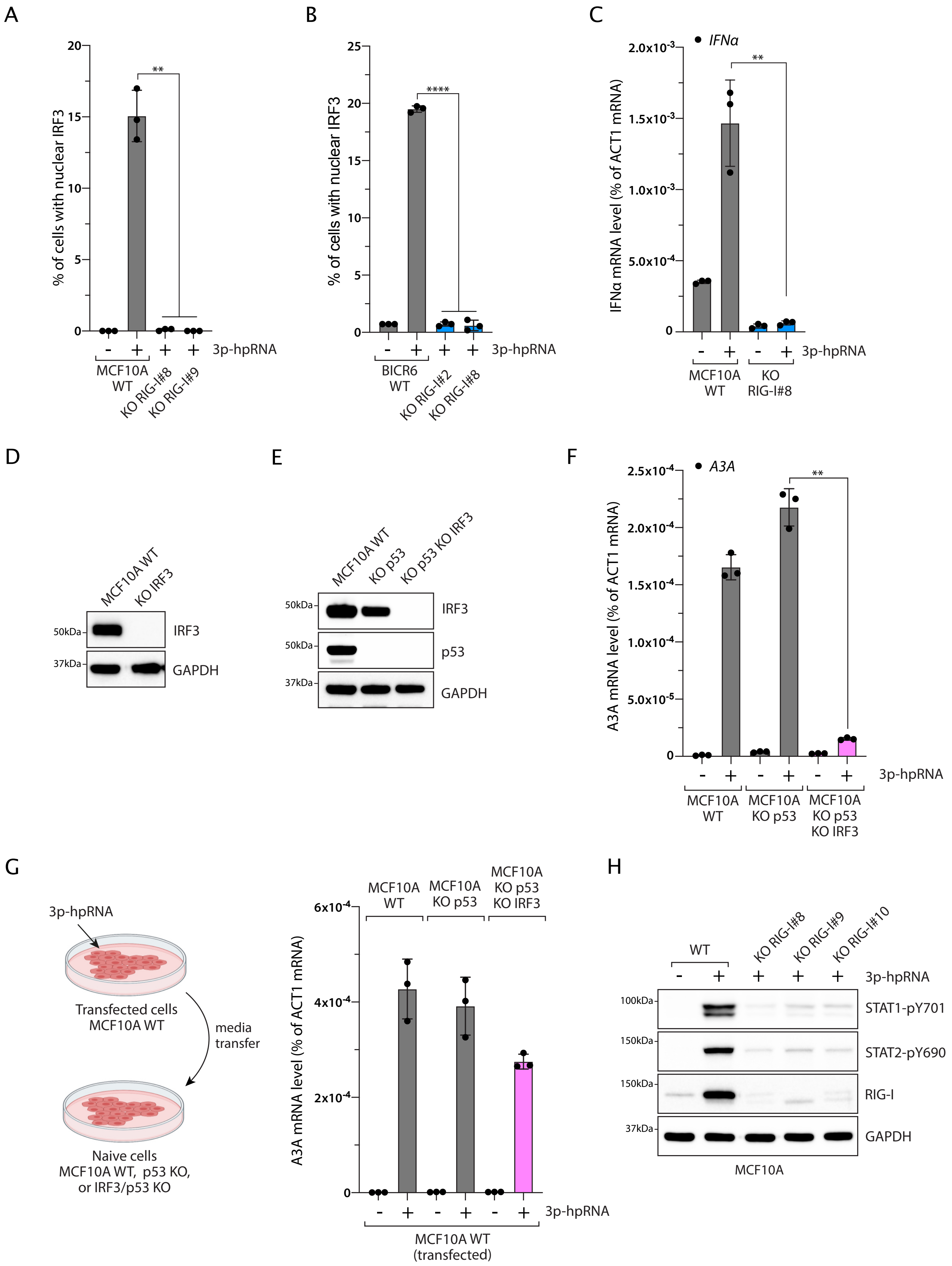

Supplementary Figure 3: A-B. Quantification of nuclear IRF3 in the indicated cell lines 16h after 3p-hpRNA transfection (100 ng/mL). Mean values +/- S.D. (n = 3). ** $P < 0.01$, **** $P < 0.0001$ (two-tailed Welch t test). **C.** Quantification of IFNα mRNA level by RT-qPCR in MCF10A WT cells and RIG-I KO cells transfected with 100 ng/mL of 3p-hpRNA for 16h. Mean values +/- S.D. (n = 3). ** $P < 0.01$ (two-tailed Welch t test). **D-E.** Indicated cell lysates were analyzed by Western blot. **F.** The A3A mRNA level was monitored in the indicated cell lines transfected with 3p-hpRNA for 16h. Mean values +/- S.D. (n = 3). ** $P < 0.01$ (two-tailed Welch t test). **G.** MCF10A cells were transfected with 3p-hpRNA for 8h following by 16h incubation in fresh media. Then, the conditioned media was collected, filtered, and then added to indicated naïve cells for 24h. The A3A mRNA level was quantify by RT-qPCR. Mean values +/- S.D. (n = 3). **H.** MCF10A WT cells or RIG-I KO cells were transfected with 3p-hpRNA (100 ng/mL) for 16h before collection for Western Blot analysis using indicated antibodies. Source data are provided as a Source Data file.

Supplementary Figure 4

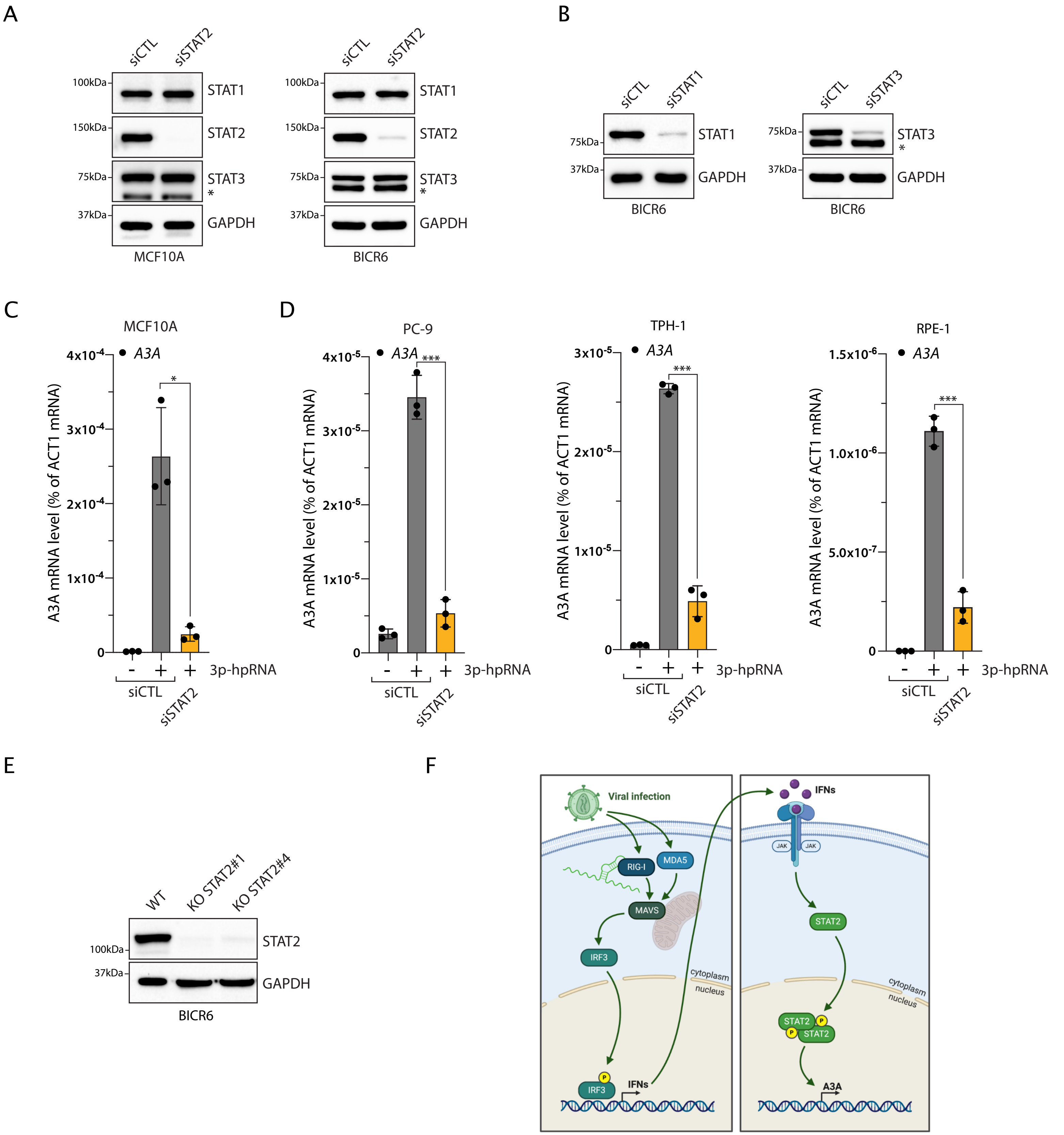

Supplementary Figure 4: A-B. MCF10A and BICR6 cells were transfected with control, STAT1, STAT2, or STAT3 siRNAs. Cell lysates were analyzed by Western blot using the indicated antibodies. **C-D.** MCF10A, PC-9, TPH-1, and RPE-1 cells were transfected with STAT2 siRNA for 36h followed by 3p-hpRNA transfection (100ng/mL) for 16h. A3A expression level was determined by RT-qPCR. Mean values +/- S.D. (n = 3). * P <0.05, *** P < 0.001 (two-tailed Welch t test). **E.** BICR6 WT and STAT2 KO cell lysates were analyzed by Western Blot using the indicated antibodies. **F.** A model of A3A expression regulation by viral infection through the activation of RIG-I/MAVS signaling pathway leading to the production of IFNs and the stimulation of STAT2. Source data are provided as a Source Data file.

Supplementary Figure 5

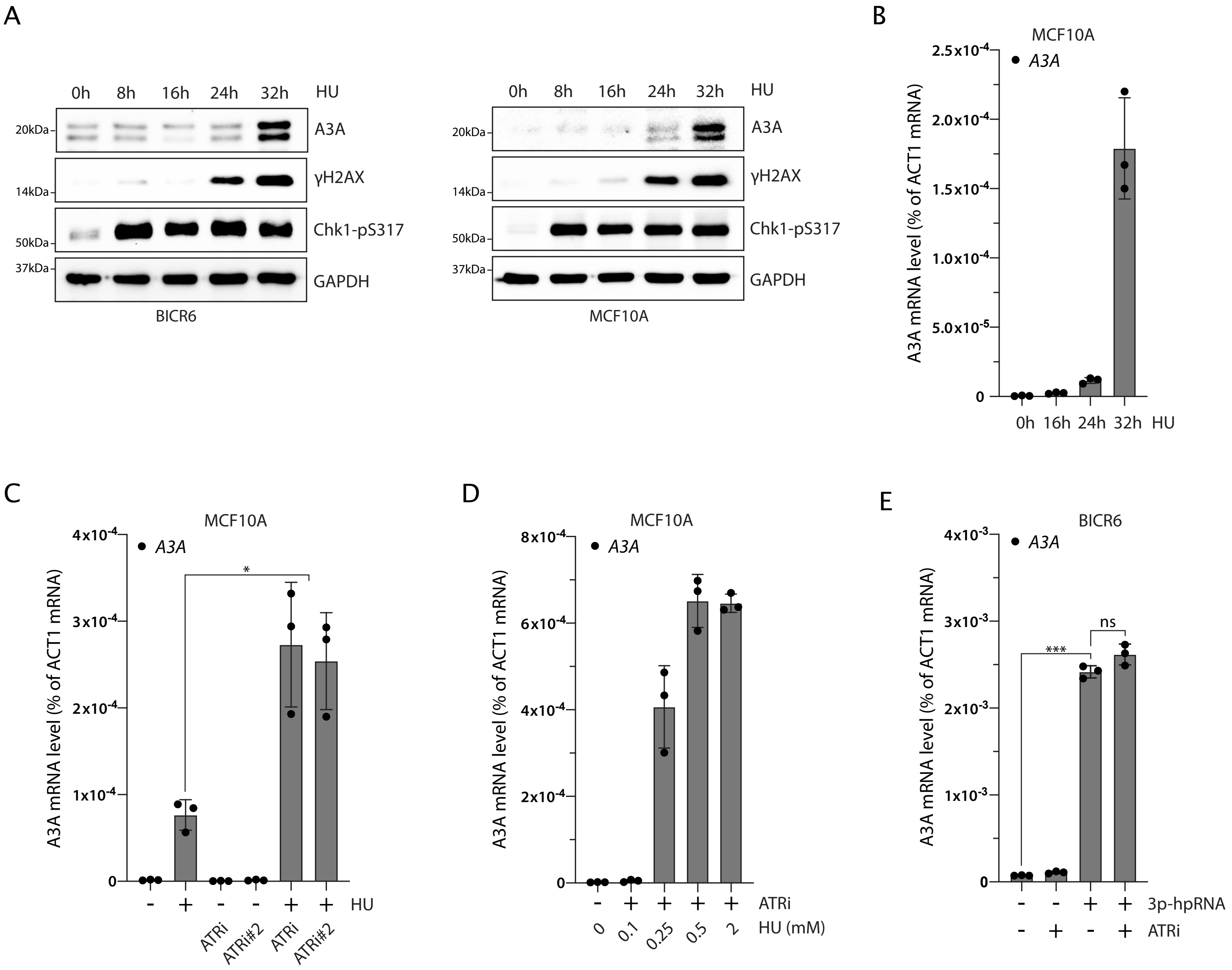

Supplementary Figure 5: A. BICR6 and MCF10A cells were treated with HU for the indicated time. Cell lysates were analyzed by Western Blot using the indicated antibodies. **B.** MCF10A cells were treated with HU (2 mM) and A3A mRNA level was analyzed at the indicated times. Mean values +/- S.D. (*n* = 3). **C.** MCF10A cells were treated with HU (2 mM), ATRi (1 μM; VE-821), ATRi#2 (0.5 μM; AZD6738) or the combinations of these drugs for 32h and the level of A3A was monitored by RT-qPCR. Mean values +/- S.D. (n = 3). * *P* <0.05 (two-tailed Welch t test). **D.** MCF10A cells were treated with ATRi (1 μM; VE-821) and an increasing concentration of HU. The level of A3A mRNA was quantify by RT-PCR. Mean values +/- S.D. (n = 3). **E.** Quantification of A3A mRNA level by RT-qPCR in BICR6 cells transfected with 100 ng/mL of 3p-hpRNA for 16h and /or treated with ATRi for 32h. Mean values +/- S.D. (n = 3). ns: not significant (*P* > 0.05), *** *P* < 0.001 (two-tailed Welch t test). Source data are provided as a Source Data file.

Supplementary Figure 6

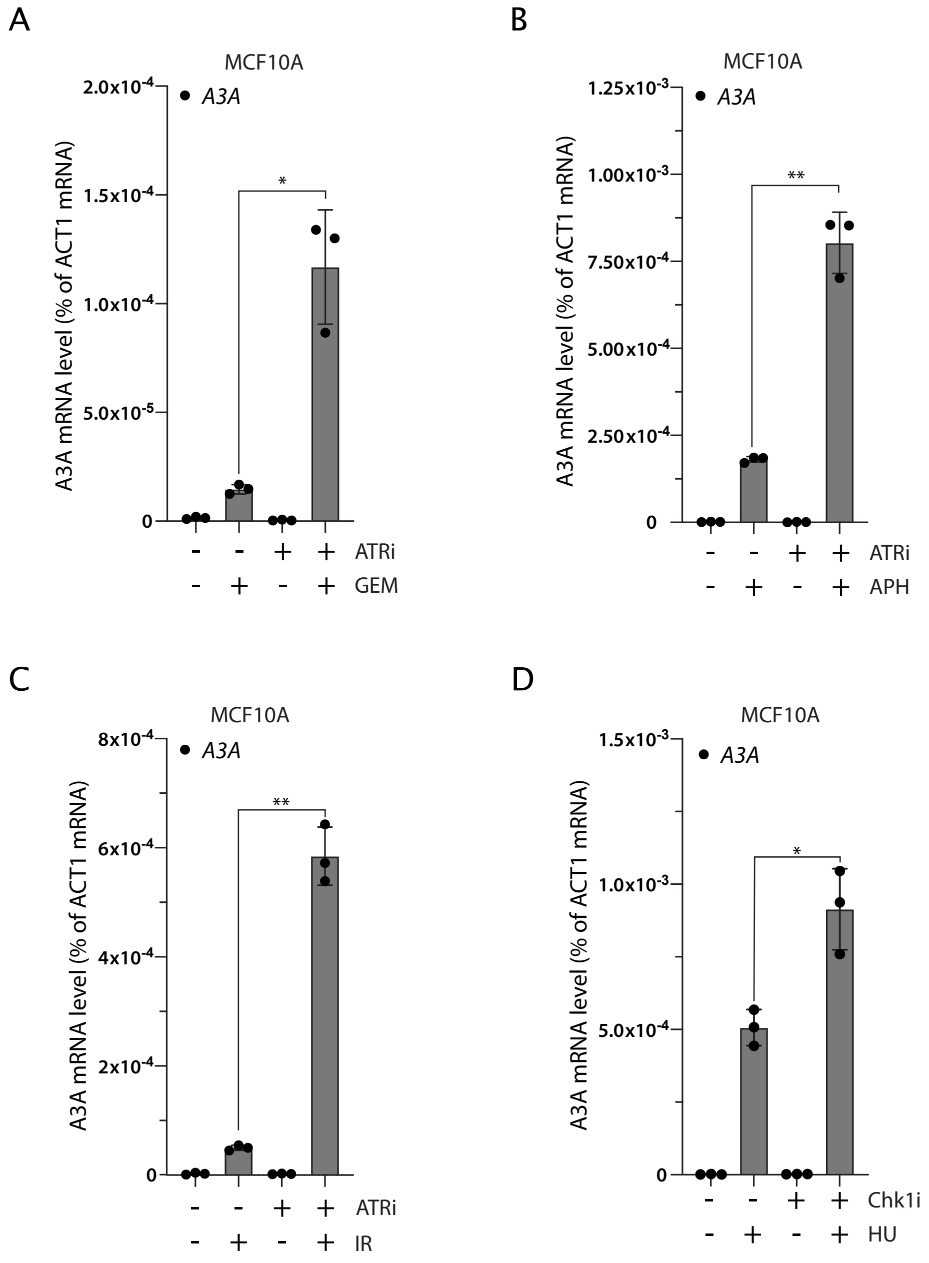

Supplementary Figure 6: A-C. MCF10A cells were treated with GEM (0.5 µM for 32h), APH (0.5 µg/mL for 32h), or IR (10 Gy for 72h), in combinations with ATRi (1 µM; VE-821). A3A mRNA level was monitored by RT-qPCR. Mean values +/- S.D. (n = 3). * P <0.05, ** P < 0.01 (two-tailed Welch t test). **D.** A3A expression was analyzed by RT-qPCR after 32 h of HU treatment in combination with Chk1i (2 µM) in MCF10A cells. Mean values +/- S.D. (n = 3). * P <0.05 (two-tailed Welch t test). Source data are provided as a Source Data file.

Supplementary Figure 7

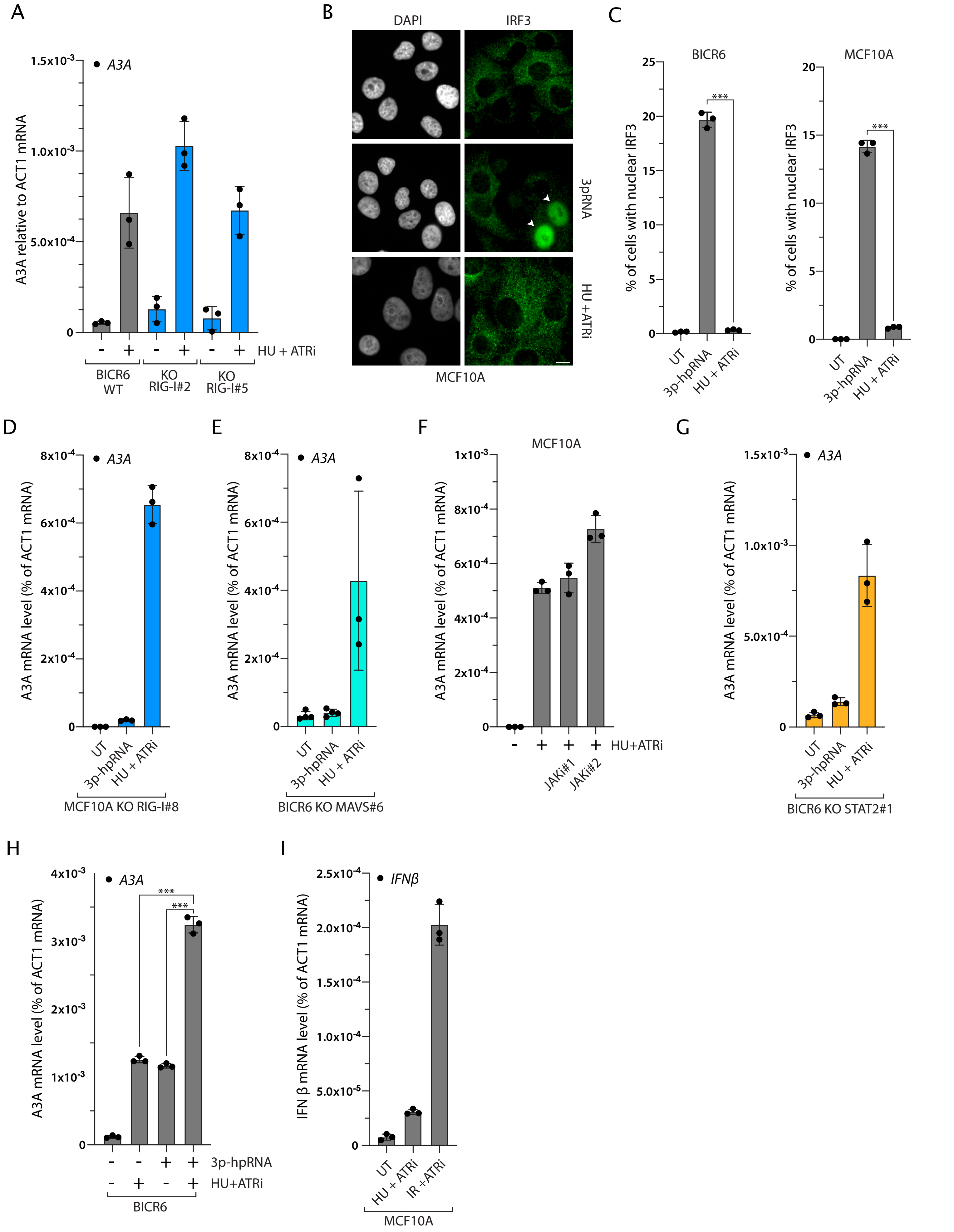

Supplementary Figure 7: A. BICR6 WT or RIG-I KO were treated for 32h with HU+ATRi and the level of A3A mRNA was monitored by RT-qPCR. Mean values +/- S.D. (n = 3). **B.** IRF3 nuclear localization was monitored by immunofluorescence in MCF10A transfected with 3p-hpRNA (16h) or treated with HU+ATRi (32h). White arrows indicate nucleus positive for IRF3. Scale bar: 10 µm. **C.** Quantification of nuclear IRF3 in BICR6 and MCF10A cells transfected with 3p-hpRNA (16h) or treated with HU+ATRi (32h). Mean values +/- S.D. (n = 3). *** $P < 0.001$ (two-tailed Welch t test). **D-E.** Indicated cell lines were treated with 3p-hpRNA or HU+ATRi for 16h and 32h respectively. A3A mRNA level was analyzed by RT-qPCR. Mean values +/- S.D. (n = 3). **F.** The A3A mRNA level was monitored 32h after treatment with JAK inhibitors (JAKi #1: 2 µM Pacritinib, JAKi #2: 2 µM Ruxolitinib) and HU in MCF10A cells. Mean values +/- S.D. (n = 3). **G.** STAT2 KO cells were treated with 3p-hpRNA or HU+ATRi for 16h and 32h respectively. A3A mRNA levels were analyzed by RT-qPCR. Mean values +/- S.D. (n = 3). **H.** The A3A mRNA levels were monitored by RT-qPCR in BICR6 cells following indicated treatments. *** $P < 0.001$ (two-tailed Welch t test). **I.** MCF10A cells were treated with HU+ATRi (32h) or IR+ATRi (72h). IFNβ mRNA levels were analyzed by RT-qPCR. Mean values +/- S.D. (n = 3). Source data are provided as a Source Data file.

Supplementary Figure 8

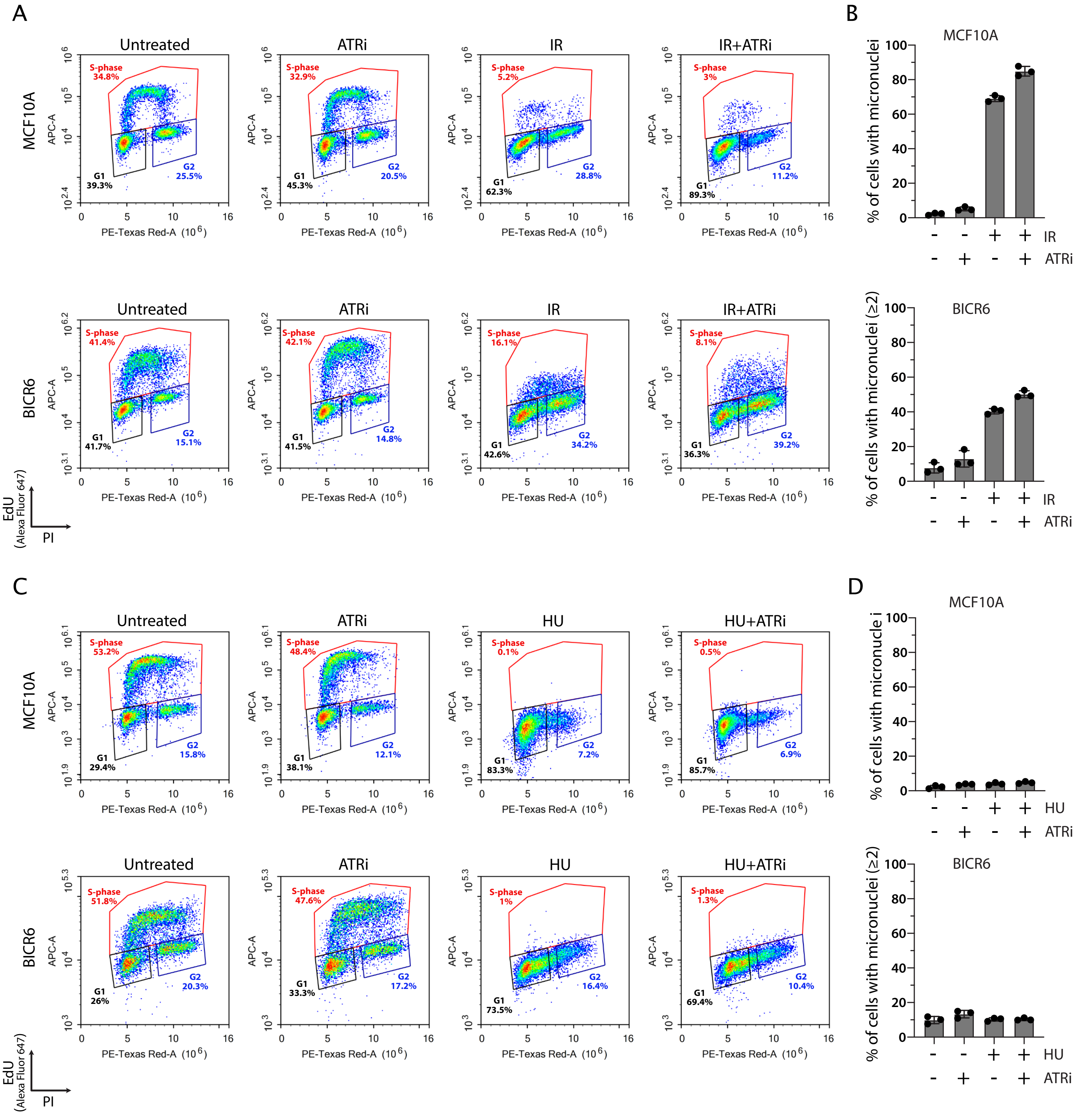

Supplementary Figure 8: A. MCF10A and BICR6 cell cycle profiles monitored by flow cytometry following indicated treatments. MCF10A and BICR6 cells were incubated for 30min with EdU (10 μM), 71.5h after IR, ATRi or IR+ATRi. **B.** Micronuclei quantification in MCF10A and BICR6 cells treated with IR, ATRi or IR+ATRi for 72h. Mean values +/- S.D. (n = 3). **C.** MCF10A and BICR6 cell cycle profiles monitored by flow cytometry following indicated treatments. MCF10A and BICR6 cells were incubated for 30min with EdU (10 μM), 31.5h after HU, ATRi or HU+ATRi. **D.** Micronuclei quantification in MCF10A and BICR6 cells treated with HU, ATRi or HU+ATRi for 32h. Mean values +/- S.D. (n = 3). Source data are provided as a Source Data file.

Supplementary Figure 9

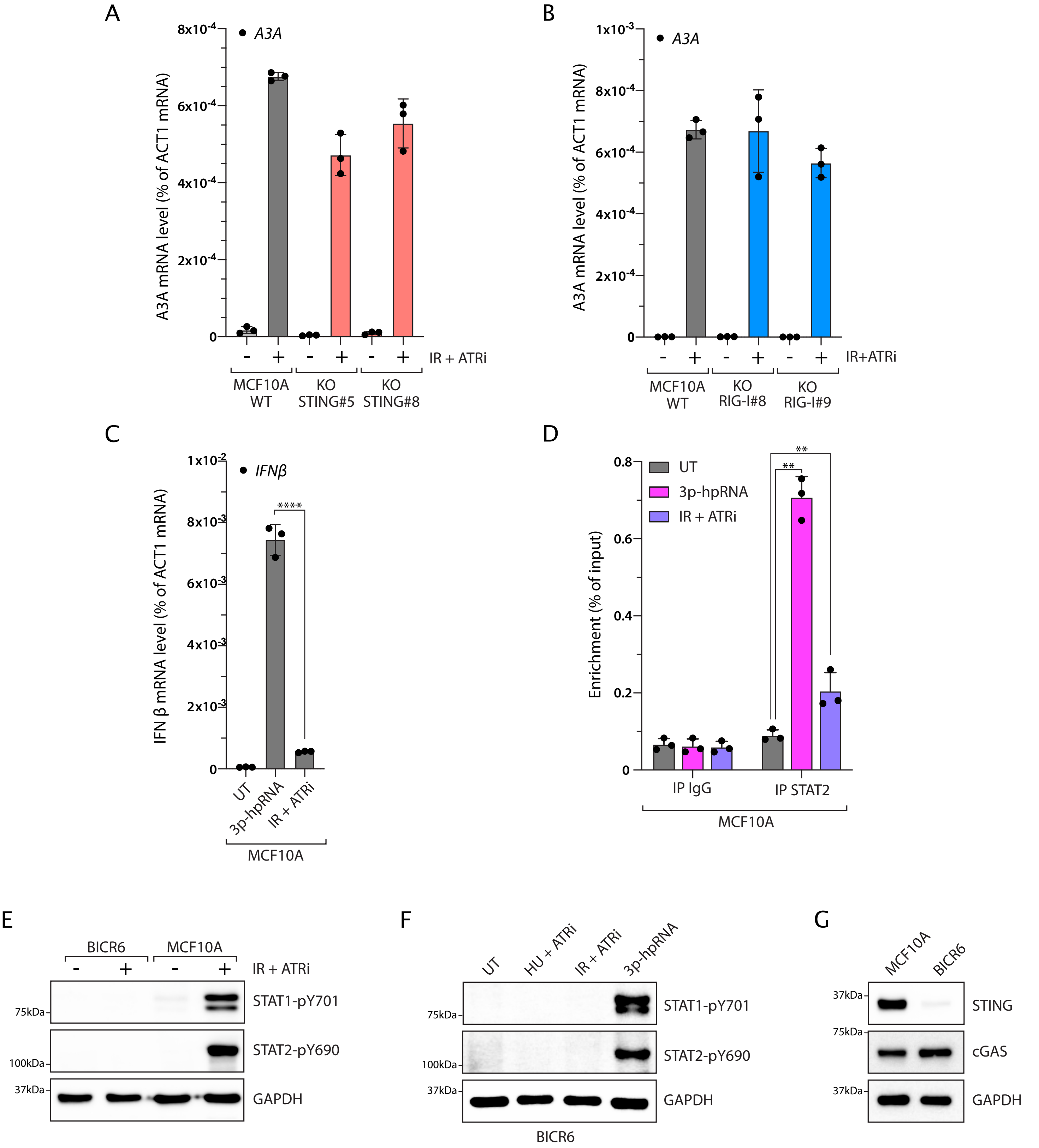

Supplementary Figure 9: A-B. Indicated cell lines were treated with IR+ATRi for 72h and A3A expression level was determined by RT-qPCR. Mean values +/- S.D. (n = 3). **C.** MCF10A cells were treated with 3p-hpRNA (16h) or IR+ATRi (72h). IFNβ mRNA levels were analyzed by RT-qPCR. Mean values +/- S.D. (n = 3). **** $P < 0.0001$ (two-tailed Welch t test). **D.** STAT2 ChIP was performed in MCF10A cells transfected with 3p-hpRNA for 16h or IR+ATRi for 72h. STAT2 binding at the A3A promoter was determined by qPCR. Mean values +/- S.D. (n = 3). ** $P < 0.01$ (two-tailed Welch t test). **E-F.** STAT1-pY701 and STAT2-pY690 levels were monitored by western blot in BICR6 or MCF10A cells following indicated treatments. **G.** STING and cGAS protein levels were monitored by western blot in both MCF10A and BICR6. Source data are provided as a Source Data file.

Supplementary Figure 10

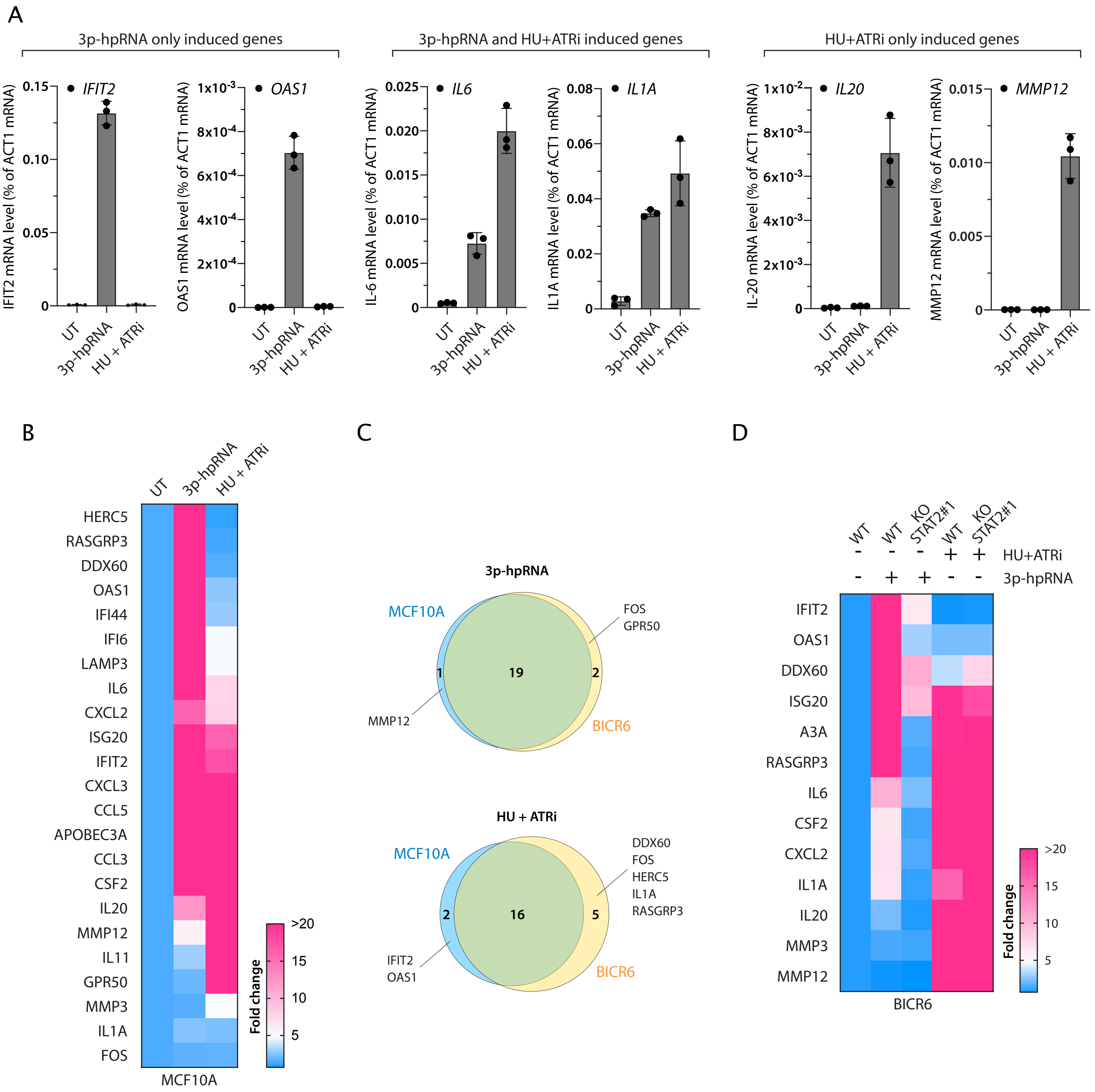

Supplementary Figure 10: A. Indicated genes expression levels were determined by RT-qPCR in BICR6 cells treated with HU+ATRi or 3p-hpRNA. Mean values +/- S.D. (n = 3). **B.** Heat maps of inflammatory genes expression level in MCF10A cells treated with 3p-hpRNA or HU+ATRi. **C.** Venn diagram to illustrate differential gene expression between MCF10A and BICR6 cells after 3p-hpRNA transfection (top) or HU+ATRi treatment (bottom). Only genes from the heat maps of Figure 6C and Supplementary Figure 10B that are up or downregulated with a fold change >2 are shown. **D.** Heat maps of inflammatory gene mRNA levels in BICR6 WT or KO STAT2 after treatment with 3p-hpRNA or HU+ATRi. Source data are provided as a Source Data file.

Supplementary Figure 11

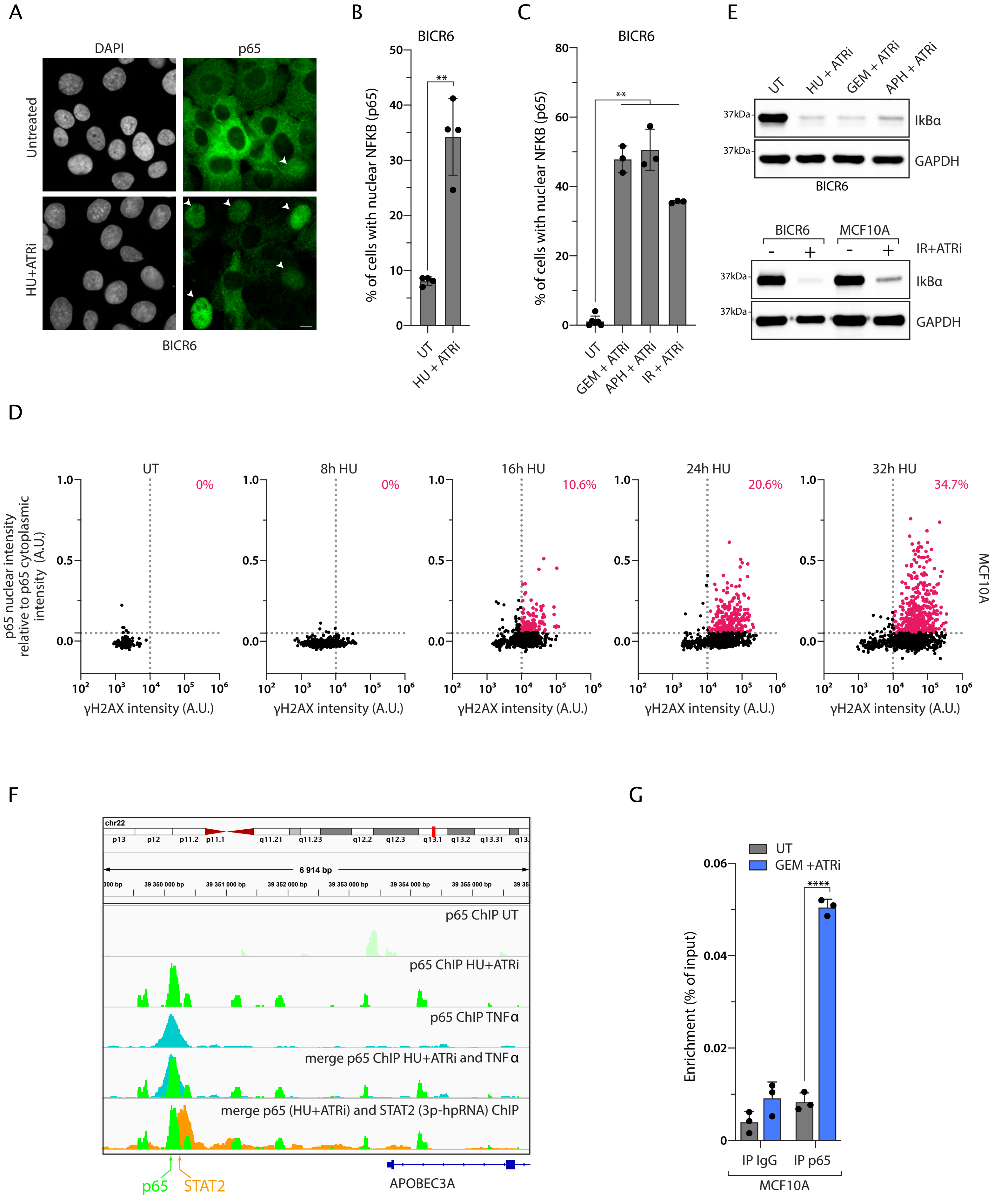

Supplementary Figure 11: A. p65 localization in BICR6 cells after 32h of HU+ATRi treatment. White arrows indicate nucleus positive for p65. Scale bar: 10 µm. **B-C.** Quantification of nuclear p65 in BICR6 cells treated with the indicated treatment. Mean values +/- S.D. (n = 3). ** $P < 0.01$ (two-tailed Welch t test). **D.** The levels of γH2AX and p65 signals were quantified in MCF10A cells treated with HU for the indicated time. **E.** Top: BICR6 cells were treated with the indicated treatment for 32h. Bottom: BICR6 or MCF10A cells were treated with IR+ATRi for 72h. The level of IkBα was analyzed by Western Blotting. **F.** Analysis of p65 ChIP-sequencing data after indicated treatment in MCF10A cells with a focus on the region upstream of A3A TSS. Middle panels: Sequencing result after TNFα treatment was obtained from the analysis of previously published p65 ChiP sequencing data (GSM935478). Bottom panel: STAT2 ChiP sequencing result after 3p-hpRNA transfection showed in Figure 3F was overlaid to the p65 ChiP sequencing result after HU+ATRi treatment. Arrows indicate the predicted p65 and STAT2 binding sites. **G.** p65 ChIP was performed in MCF10A cells treated with GEM+ATRi for 32h. p65 binding at A3A promoter was determined by qPCR. The results are representative of three independent experiments, and qPCR was done in triplicate. Mean values +/- S.D. **** $P < 0.0001$ (two-tailed Welch t test). Source data are provided as a Source Data file.

Supplementary Figure 12

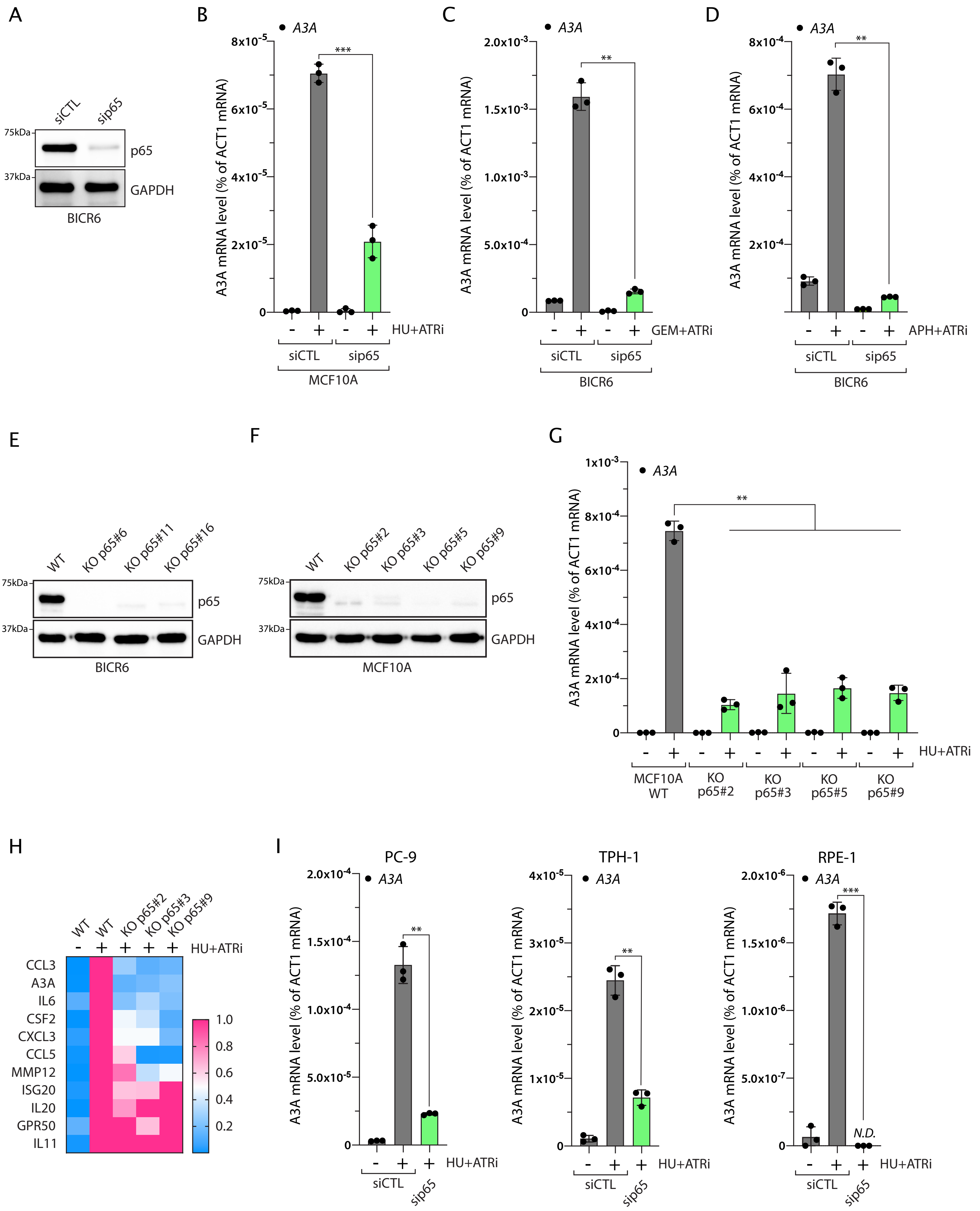

Supplementary Figure 12: A. BICR6 cells were transfected with control or p65 siRNA. Cell lysates were analyzed by Western blot using the indicated antibodies. **B.** MCF10A cells were first transfected with p65 siRNA for 40h and then treated with HU+ATRi for 32h. A3A expression level was determined by RT-qPCR. Mean values +/- S.D. (n = 3). *** $P < 0.001$ (two-tailed Welch t test). **C-D.** BICR6 cells were transfected with control or p65 siRNA and then treated with the indicated drugs. A3A expression level was determined by RT-qPCR. Mean values +/- S.D. (n = 3). ** $P < 0.01$ (two-tailed Welch t test). **E-F.** BICR6 or MCF10A WT and p65 KO cell lysates were analyzed by Western Blot using the indicated antibodies. **G.** A3A mRNA level quantification after HU+ATRi for 32h in MCF10A wild type or knockout for p65. Mean values +/- S.D. (n = 3). ** $P < 0.01$ (two-tailed Welch t test). **H.** Heat maps of inflammatory genes expression level in MCF10A WT or p65 KO treated with HU+ATRi. **I.** PC-9, TPH-1, or RPE-1 cells were first transfected with p65 siRNA for 40h and then treated with HU+ATRi for 32h. A3A expression level was determined by RT-qPCR. Mean values +/- S.D. (n = 3). N.D; Not Detected. ** $P < 0.01$, *** $P < 0.001$ (two-tailed Welch t test). Source data are provided as a Source Data file.

Supplementary Figure 13

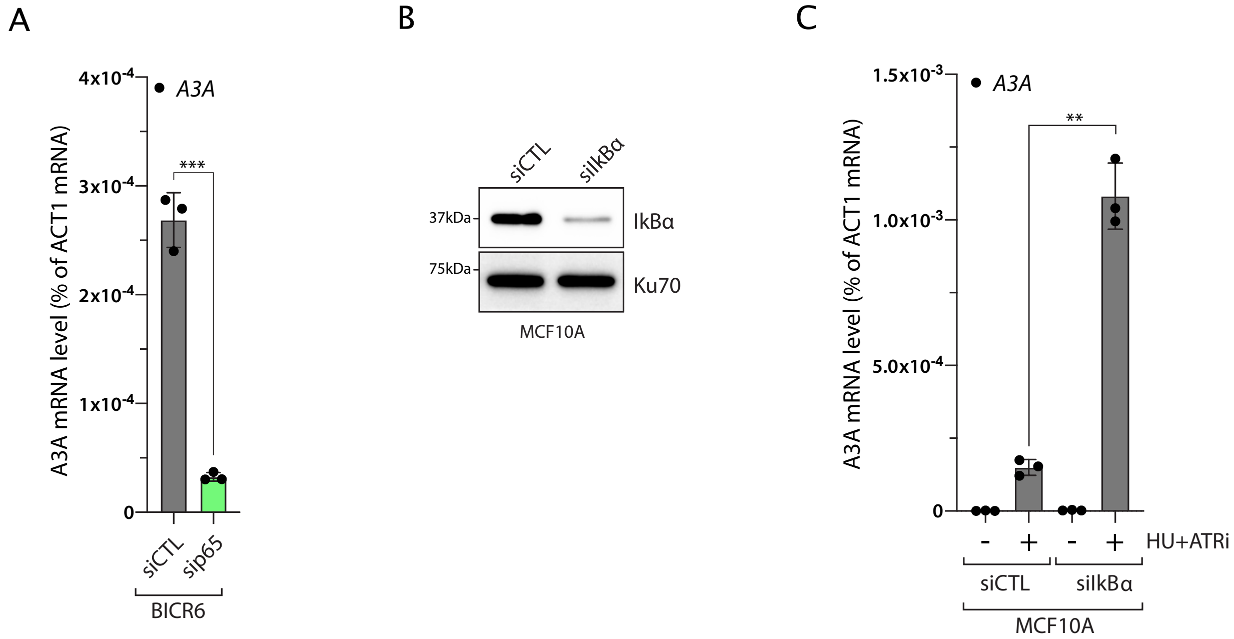

Supplementary Figure 13: A. BICR6 cells were first transfected with p65 siRNA for 40h and the A3A mRNA was quantified by RT-qPCR. Mean values +/- S.D. (n = 3). *** $P < 0.001$ (two-tailed Welch t test). **B.** BICR6 cells were transfected with control or p65 siRNA. Cell lysates were analyzed by Western blot using the indicated antibodies. **C.** MCF10A cells were transfected with IkBα siRNA for 40h following by HU+ATRi treatment for 32h. A3A expression level was determined by RT-qPCR. Mean values +/- S.D. (n = 3). ** $P < 0.01$ (two-tailed Welch t test). Source data are provided as a Source Data file.

Targets	Sequences
siCTL (#4390846)	Not disclosed by the company
siMDA5 (#s34500)	GUUCAGGAGUUAUCGAACAtt
siSTAT1 (#s279)	GGUUCACUAUAGUUGCGGAtt
siSTAT2 (#s13528)	GGCUCAUUGUGGUCUCUAAtt
siSTAT3 (#s744)	GGCUGGACAAUAUCAUUGAtt
siIkBα (#s9512)	AGUUCACGGAGUUCACAGAtt
sip65 (#s11915)	GGAGUACCCUGAGGCUAUAtt
siA3A	CGACAGUACCAGACUCCAUtt

Supplementary Table 1: RNA interference sequences.

Targets	Sequences
RIG-I gRNA	GGATTATATCCGGAAGACCC
STING gRNA	TACTCCCTCCCAAATGCGGT
STAT2 gRNA	AAGTACTGTCGAATGTCCAC
p65 gRNA	GCTCAATGATCTCCACATAG
MAVS gRNA#1	GACAGGGTCAGTTGTATCTAC
MAVS gRNA#2	GCAACTCAACCCGTGCTGGCA
IRF3 gRNA#1	TTGGAAGCACGGCCTACGGC
IRF3 gRNA#2	GGGTGAACAAGAGCCGCACG
IRF3 gRNA#3	CAACCGCAAAGAAGGGTTGC

Supplementary Table 2: Guide RNA sequences.

Targets	Species and Antibody types	Company and Catalogue number	Antibody Dilution
GAPDH	Rabbit polyclonal	EMD Millipore (#ABS16)	1/20,000 WB
Ku70	Mouse monoclonal	GeneTex (#GTX70271)	1/5000 WB
MDA5	Rabbit monoclonal	Cell signaling (#5321)	1/1000 WB
RIG-I	Rabbit monoclonal	Cell signaling (#3743)	1/1000 WB
STING	Rabbit monoclonal	Cell Signaling (#13647)	1/1000 WB
APOBEC3A	Rabbit monoclonal	NIH-ARP (#12398)	1/1000 WB
STAT1	Mouse monoclonal	Santa Cruz (#sc-464)	1/1000 WB
STAT2	Rabbit monoclonal	Cell Signaling (#72604)	1/1500 WB
STAT3	Rabbit polyclonal	Bethyl (#A302-405A-T)	1/2000 WB
STAT2-pY690	Rabbit monoclonal	Cell Signaling (#88410)	1/1500 WB
STAT1-pY701	Rabbit monoclonal	Cell Signaling (#9167)	1/1000 WB
γH2AX	Mouse monoclonal	EMD Millipore (#JBW301)	1/5000 WB; 1/1000 IF
CHK1-pS345	Rabbit monoclonal	Cell Signaling (#2348)	1/1000 WB
CHK1-pS317	Rabbit polyclonal	Cell Signaling (#2344)	1/1000 WB
IRF3	Rabbit monoclonal	Cell Signaling (#11904)	1/500 WB; 1/300 IF
NFκB (p65)	Mouse monoclonal	Santa Cruz (#sc8008)	1/400 IF
NFκB (p65)	Rabbit monoclonal	Cell Signaling (#8242)	1/2000 WB; 1/500 IF
IkBα	Mouse monoclonal	Cell Signaling (#4814)	1/1000 WB
p53	Mouse monoclonal	Santa Cruz (#sc-47698)	1/500 WB
cGAS	Rabbit monoclonal	Cell Signaling (#15102)	1/1000 WB

Supplementary Table 3: Antibodies. WB: Western Blotting, IF: Immunofluorescence.

Primer name	Forward	Reverse
Actin	CCAACCGCGAGAAGATGA	CCAGAGGCGTACAGGGATAG
APOBEC3A	GAGAAGGGACAAGCACATGG	TGGATCCATCAAGTGTCTGG
APOBEC3B	GACCCTTTGGTCCTTCGAC	GCACAGCCCCAGGAGAAG
APOBEC3C	AGCGCTTCAGAAAAGAGTGG	AAGTTTCGTTCCGATCGTTG
APOBEC3D	ACCCAAACGTCAGTCGAATC	CACATTTCTGCGTGGTTCTC
APOBEC3F	CCGTTTGGACGCAAAGAT	CCAGGTGATCTGGAAACACTT
APOBEC3G	CCGAGGACCCGAAGGTTAC	TCCAACAGTGCTGAAATTCG
APOBEC3H	AGCTGTGGCCAGAAGCAC	CGGAATGTTTCGGCTGTT
IFN-β	ACACTGGTCGTGTTGTTGAC	GGAAAGAGCTGTCGTGGAGA
IFN-α	GTGAGGAAATACTTCCAAAGAATCAC	TCTCATGATTTCTGCTCTGACAA
IL6	CAGCCCTGAGAAAGGAGACAT	GGTTCAGGTTGTTTTCTGCCA
IFIT2	TGGTGGCAGAAGAGGAAGAT	GTAGGCTGCTCTCCAAGGAA
OAS1	GTGTGTCCAAGGTGGTAAAGG	CTGCTCAAACTTCACGGAA
IFI44	CCACCGAGATGTCAGAAAGAG	TGGTACATGTGGCTTTGCTC
DDX60	AAGGTGTTCCTTGATGATCTCC	TGACAATGGGAGTTGATATTCC
IFI6	CTGGTCTGCGATCCTGAATG	AGAGGTTCTGGGAGCTGCTG
LAMP3	TGAAAACAACCGATGTCCAA	TCAGACGAGCACTCATCCAC
ISG20	TGGACTGCGAGATGGTGG	GGGTTCTGTAATCGGTGAT
HERC5	GATTGCTGGAGGGAATCAAA	TTGGATTTCCCTTTTTGTGC
IL1A	ATCAGTACCTCACGGCTGCT	TGGGTATCTCAGGCATCTCC
CCL5	ACACCCTGCTGCTTTGCCTACA	TCCCGAACCCATTTCTTCTCTG
RASGRP3	GGGAAAAGCCTGTCTGCTGTT	GCTCCAGAAAAGTGAGGTGCT
CCL3	TCTGCATCACTTGCTGCTGACAC	CACTCAGCTCCAGGTCGCTGAC
IL6	CAGCCCTGAGAAAGGAGACAT	GGTTCAGGTTGTTTTCTGCCA
CXCL2	CATCGAAAAGATGCTGAAAAATG	TTCAGGAACAGCCACCAATA
CSF2	CACTGCTGCTGAGATGAATGAAA	GTCTGTAGGCAGGTCGGCTC
IL11	TCTCTCCTGGCGGACACG	AATCCAGGTTGTGGTCCCC
CXCL3	AAAATCATCGAAAAGATACTGAACAAG	GTAAGGGCAGGGACCAC
GPR50	CTTTGATGCTGCATGCCATGTCCA	TGTGGCAGATGTAGCAGTAACGGT
IL20	CAAGACACAAAGCCTGCGAATC	CTTCATTGCTTCCTCCCCACA
FOS	CCAACCTGCTGAAGGAGAAG	AGATCAAGGGAAGCCACAGA
MMP12	TGGTTTTTGCCCGTGGAGCTCAT	GAATGGCCAATCTCGTGAACAGCA
MMP3	TCATTTTGGCCATCTCTTCC	CCAGCTCGTACCTCATTTCC

Supplementary Table 4: qPCR primer sequences.

Primer name	Forward	Reverse
A3A region 1 (p65)	CCCAGACACACCCAGATGTC	TGATGACGTGGTTTCCCCAG
A3A region 2 (STAT2)	CATCAACCCCGATCCTCTCG	TGTTTCAAGGGGTGGAGCTG

Supplementary Table 5: ChiP-qPCR primer sequences.
