# Supplementary material for: Genotoxic stress and viral infection induce transient expression of APOBEC3A and pro-inflammatory genes through two distinct pathways
Source: Nat Commun. 2021 Aug 13;12:4917. doi: 10.1038/s41467-021-25203-4 (PMC8363607; doi:10.1038/s41467-021-25203-4)
Supplement: Supplementary file 3 — Description of Additional Supplementary Files [file 41467_2021_25203_MOESM3_ESM.pdf]

Description of Additional Supplementary Files

File Name: Supplementary Data 1
Description: **List of differentially expressed genes in BICR6 cells treated with 3p-hpRNA or HU+ATRi.** log2(fold change) and p-value (-log10) are indicated for each listed genes after indicated treatment. Colored p-values indicate genes with log2(fold change) >2. P-values were calculated for associated logistic regressions between gene expression levels and treatment condition using DESeq2 and P-values of genes expressed after both treatments are colored in red.
