# Supplementary material for: Genotoxic stress and viral infection induce transient expression of APOBEC3A and pro-inflammatory genes through two distinct pathways
Source: Nat Commun. 2021 Aug 13;12:4917. doi: 10.1038/s41467-021-25203-4 (PMC8363607; doi:10.1038/s41467-021-25203-4)
Supplement: Supplementary file 5 — Reporting Summary [file 41467_2021_25203_MOESM5_ESM.pdf]

nature research

Corresponding author(s): Rémi Buisson

Last updated by author(s): Jun 11, 2021

Reporting Summary

Nature Research wishes to improve the reproducibility of the work that we publish. This form provides structure for consistency and transparency in reporting. For further information on Nature Research policies, see our Editorial Policies and the Editorial Policy Checklist.

Statistics

For all statistical analyses, confirm that the following items are present in the figure legend, table legend, main text, or Methods section.

n/a	Confirmed	
☐	☒	The exact sample size (*n*) for each experimental group/condition, given as a discrete number and unit of measurement
☐	☒	A statement on whether measurements were taken from distinct samples or whether the same sample was measured repeatedly
☐	☒	The statistical test(s) used AND whether they are one- or two-sided<br>*Only common tests should be described solely by name; describe more complex techniques in the Methods section.*
☒	☐	A description of all covariates tested
☒	☐	A description of any assumptions or corrections, such as tests of normality and adjustment for multiple comparisons
☐	☒	A full description of the statistical parameters including central tendency (e.g. means) or other basic estimates (e.g. regression coefficient) AND variation (e.g. standard deviation) or associated estimates of uncertainty (e.g. confidence intervals)
☐	☒	For null hypothesis testing, the test statistic (e.g. *F*, *t*, *r*) with confidence intervals, effect sizes, degrees of freedom and *P* value noted<br>*Give P values as exact values whenever suitable.*
☒	☐	For Bayesian analysis, information on the choice of priors and Markov chain Monte Carlo settings
☒	☐	For hierarchical and complex designs, identification of the appropriate level for tests and full reporting of outcomes
☒	☐	Estimates of effect sizes (e.g. Cohen's *d*, Pearson's *r*), indicating how they were calculated

Our web collection on statistics for biologists contains articles on many of the points above.

Software and code

Policy information about availability of computer code

Data collection	qRT–PCR was performed in QuantStudio 3 (Applied Biosystems) using Applied Biosystems QuantStudio software.<br>FACS data acquisition was performed on a NovoCyte Flow cytometer equipped with the NovoExpress software (ACEA Biosciences)<br>Gel images were acquired using a Chemidoc MP (BioRad).
Data analysis	Gel images were quantified with ImageLab 6.1 (Biorad).<br>Graphs were generated using Prim 9 (GraphPad).<br>FACS data analysis was performed using NovoExpress software (ACEA Biosciences)

For manuscripts utilizing custom algorithms or software that are central to the research but not yet described in published literature, software must be made available to editors and reviewers. We strongly encourage code deposition in a community repository (e.g. GitHub). See the Nature Research guidelines for submitting code & software for further information.

Data

Policy information about availability of data

All manuscripts must include a data availability statement. This statement should provide the following information, where applicable:

- Accession codes, unique identifiers, or web links for publicly available datasets
- A list of figures that have associated raw data
- A description of any restrictions on data availability

The source data underlying Figures 1 to 7, and Supplementary Figure 1 to 13 are provided as a Source Data file. All sequencing data were deposited in NCBI Sequence Read Archive, using the Bioproject Accession: PRJNA684601. A direct link can be found here: https://www.ncbi.nlm.nih.gov/bioproject/?term=PRJNA684601. Databases used in this study were: Stanford_ChipSeq_GM12878_TNFa_NFKB_IgG-rab (https://www.ncbi.nlm.nih.gov/geo/query/acc.cgi?acc=GSM935478) and the human genome GRCh37/hg19 (https://www.ncbi.nlm.nih.gov/assembly/GCF_000001405.13/) and GRCh38/hg19 (https://

www.ncbi.nlm.nih.gov/assembly/GCF_000001405.26/).

Field-specific reporting

Please select the one below that is the best fit for your research. If you are not sure, read the appropriate sections before making your selection.

☒ Life sciences ☐ Behavioural & social sciences ☐ Ecological, evolutionary & environmental sciences

For a reference copy of the document with all sections, see nature.com/documents/nr-reporting-summary-flat.pdf

Life sciences study design

All studies must disclose on these points even when the disclosure is negative.

Sample size	Sample sizes were chosen according to accepted standards in the field. Sample size was not pre-determined using statistics tolls. As indicated in the figure legends, minimal size of analyzed biological samples was "3". Statistical analysis (as described in respective figure legends) was used to calculate statistical significance of obtained results. The individual p-values are indicated in figures or in figure legends.
Data exclusions	No data were excluded.
Replication	All experiments have been repeated in multiple successfully independent experiments (3 times or more). All key experiments have been repeated in different cell lines.
Randomization	We had a limited number of biological samples. The analysis was self-normalized to the sample, so randomization of samples would not be a relevant method.
Blinding	As the analysis required comparisons against a known controls and knockdown targets were selected for their likely relevance to the biological pathway, blinding would not provide much reduction of potential bias in the analysis. However, performance and analyses of experiments were independently conducted by co-authors.

Reporting for specific materials, systems and methods

We require information from authors about some types of materials, experimental systems and methods used in many studies. Here, indicate whether each material, system or method listed is relevant to your study. If you are not sure if a list item applies to your research, read the appropriate section before selecting a response.

Materials & experimental systems

n/a	Involved in the study	
☐	☒	Antibodies
☐	☒	Eukaryotic cell lines
☒	☐	Palaeontology and archaeology
☒	☐	Animals and other organisms
☒	☐	Human research participants
☒	☐	Clinical data
☒	☐	Dual use research of concern

Methods

n/a	Involved in the study	
☐	☒	ChIP-seq
☐	☒	Flow cytometry
☒	☐	MRI-based neuroimaging

Antibodies

Antibodies used

Targets Species and Antibody types Company and Catalogue number Antibody Dilution
GAPDH Rabbit polyclonal EMD Millipore (#ABS16) 1:20,000 WB
Ku70 Mouse monoclonal GeneTex (#GTX70271; clone 1.5) 1:5000 WB
MDA5 Rabbit monoclonal Cell signaling (#5321; clone D74E4) 1:1000 WB
RIG-I Rabbit monoclonal Cell signaling (#3743; clone D14G6) 1:1000 WB
STING Rabbit monoclonal Cell Signaling (#13647; clone 2P2F) 1:1000 WB
APOBEC3A Rabbit monoclonal NIH-ARP (#12398; clone 5210-08-15) 1:1000 WB
STAT1 Mouse monoclonal Santa Cruz (#sc-464; clone C-136) 1:1000 WB
STAT2 Rabbit monoclonal Cell Signaling (#72604; clone D9J7L) 1:1500 WB
STAT3 Rabbit polyclonal Bethyl (#A302-405A-T) 1:2000 WB
STAT2-pY690 Rabbit monoclonal Cell Signaling (#88410) 1:1500 WB
STAT1-pY701 Rabbit monoclonal Cell Signaling (#9167) 1:1000 WB
γH2AX Mouse monoclonal EMD Millipore (#05-636; clone BW301) 1:5000 WB; 1:1000 IF
CHK1-pS345 Rabbit monoclonal Cell Signaling (#2348; clone 133D3) 1:1000 WB
CHK1-pS317 Rabbit polyclonal Cell Signaling (#2344) 1:1000 WB
IRF3 Rabbit monoclonal Cell Signaling (#11904; clone D6I4C) 1:500 WB; 1:300 IF
NFκB (p65) Mouse monoclonal Santa Cruz (#sc8008; clone F-6) 1:400 IF

	NFκB (p65) Rabbit monoclonal Cell Signaling (#8242; clone D14E12) 1:2000 WB; 1:500 IF<br>IkBα Mouse monoclonal Cell Signaling (#4814; clone L35A5) 1:1000 WB<br>p53 Mouse monoclonal Santa Cruz (#sc-47698 ; clone DO-7) 1:500 WB<br>cGAS Rabbit monoclonal Cell Signaling (#15102 ; clone D1D3G) 1:1000 WB<br>Peroxidase-conjugated AffiniPure Goat Anti Rabbit (#111-035-003) or anti Mouse (#115-035-003) 1:10000 WB<br>Cy3 conjugated AffiniPure Goat Anti Rabbit (#111-165-003) or anti Mouse (#115-165-003) 1:600 IF<br>Alexa Fluor 488 conjugated AffiniPure Goat Anti Rabbit (#111-545-003) or anti Mouse (#115-545-003) 1:600 IF
Validation	Antibodies specificity against RIG-I, MAVS, IRF3, STING, p53, p65, STAT2 were confirmed in this manuscript using knockout cell lines for the corresponding protein target. Antibodies specificity against A3A, cGAS, STAT1, STAT3, MDA5, IKBα were confirmed in this manuscript using cell lines knockdown with specific siRNA for the corresponding target. CHK1-pS317, CHK1-pS345, STAT2-pY690 and STAT1-pY701 antibodies were validated by the manufacturer for western blots and previous publications (PMID: 26365377 ; PMID: 28698210). Antibodies used for loading control (GAPDH and KU70) are validated by the manufacturer.

Eukaryotic cell lines

Policy information about cell lines

Cell line source(s)	BICR6, RPE1-hTERT, PC-9, TPH-1, LLC-MK2 and MCF10A cell lines were purchased from either ATCC or Sigma-Aldrich. Knockout cell lines were derived from BICR6 or MCF10A cell lines as described in the method section.
Authentication	All cell lines were obtained from commercial repositories (ATTC, Sigma Aldrish). Upon receipt, the cell lines were expanded and frozen stocks were created. Stocks were further authenticated as follows: To identify cross-contaminated or synonymous lines, a panel of SNPs was profiled for each cell line (Sequenom) and a pair-wise comparison score was calculated. In addition, we performed short tandem repeat (STR) analysis (AmpFLSTR Identifiler, Applied Biosystems) and matched this to an existing STR profile generated by the providing repository. For the experiments described in this article, cell lines were not continuously kept in culture for more than 3 months.
Mycoplasma contamination	All cell lines were tested repetitively for Mycoplasma contamination and all cell lines were tested negative for Mycoplasma
Commonly misidentified lines<br>(See ICLAC register)	No commonly misidentified cell lines were used in the study

ChIP-seq

Data deposition

☒ Confirm that both raw and final processed data have been deposited in a public database such as GEO.

☒ Confirm that you have deposited or provided access to graph files (e.g. BED files) for the called peaks.

Data access links<br>*May remain private before publication.*	NCBI Sequence Read Archive, using the Bioproject Accession: PRJNA684601
Files in database submission	Sequencing data for all ChIP-Seq
Genome browser session<br>(e.g. UCSC)	N/A

Methodology

Replicates	One replicates per condition were performed. Sequences and peaks of interest were confirmed by ChIP-qPCR in three independent experiments.
Sequencing depth	Approx 25million reads/sample
Antibodies	STAT2 Rabbit monoclonal, Cell Signaling (#72604)<br>NFκB (p65) Rabbit monoclonal, Cell Signaling (#8242)<br>Normal Rabbit IgG, Cell Signaling (#2729)
Peak calling parameters	N/A
Data quality	Raw read quality and filtering was performed using FastQC and Picard tools, respectively. ChIP peaks were then processed using HOMER with the default parameters.
Software	Data collection and analysis were performed using HOMER tools, and Integrative Genomics Viewer was used for visualisation and panels.

Flow Cytometry

Plots

Confirm that:

☒ The axis labels state the marker and fluorochrome used (e.g. CD4-FITC).

☒ The axis scales are clearly visible. Include numbers along axes only for bottom left plot of group (a 'group' is an analysis of identical markers).

☒ All plots are contour plots with outliers or pseudocolor plots.

☒ A numerical value for number of cells or percentage (with statistics) is provided.

Methodology

Sample preparation	BICR6 and MCF10A cell lines
Instrument	ACEA NovoCyte flow cytometer
Software	ACEA NovoExpress (data collection and analysis)
Cell population abundance	10,000 single cells were quantified. Single cells were identified and isolated by gating singulets in the plot Propidium Iodide Height intensity over Propidium Iodide Area intensity.
Gating strategy	Living cells were identified and isolated by a gate in the FSC/SSC plot. Then single cells were identified and isolated by gating singulets in the plot Propidium Iodide Height intensity over Propidium Iodide Area intensity. Finally G1, S and G2/M gates were drawn on the plot representing EdU Area intensity over Propidium Iodide Area intensity.

☒ Tick this box to confirm that a figure exemplifying the gating strategy is provided in the Supplementary Information.
